# αB-crystallin stimulates VEGF secretion and tumor cell migration and correlates with enhanced distant metastasis in head and neck squamous cell carcinoma

**DOI:** 10.1186/1471-2407-13-128

**Published:** 2013-03-18

**Authors:** Chantal van de Schootbrugge, Johan Bussink, Paul N Span, Fred CGJ Sweep, Reidar Grénman, Hanneke Stegeman, Ger JM Pruijn, Johannes HAM Kaanders, Wilbert C Boelens

**Affiliations:** 1 Department of Biomolecular Chemistry, Institute for Molecules and Materials and Nijmegen Center for Molecular Life Sciences, Radboud University Nijmegen, Nijmegen, The Netherlands; 2 Department of Radiation Oncology, Radboud University Nijmegen Medical Centre, PO Box 9101, Nijmegen, 6500 HB, The Netherlands; 3 Department of Laboratory Medicine, Radboud University Nijmegen Medical Centre, PO Box 9101, Nijmegen, 6500 HB, The Netherlands; 4 Department of Otorhinolaryngology–Head and Neck Surgery, Turku University Hospital, PO Box 52, Turku, FI-20521, Finland; 5 Biomolecular Chemistry 271, NCMLS, Radboud University Nijmegen, PO Box 9101, Nijmegen, 6500 HB, The Netherlands

**Keywords:** CRYAB protein, HspB5, Vascular endothelial growth factor A, Cell movement, Neoplasm metastasis, Carcinoma, Squamous cell of head and neck

## Abstract

**Background:**

αB-crystallin is able to modulate vascular endothelial growth factor (VEGF) secretion. In many solid tumors VEGF is associated with angiogenesis, metastasis formation and poor prognosis. We set out to assess whether αB-crystallin expression is correlated with worse prognosis and whether this is related to VEGF secretion and cell motility in head and neck squamous cell carcinoma (HNSCC).

**Methods:**

αB-crystallin expression was determined immunohistochemically in tumor biopsies of 38 HNSCC patients. Locoregional control (LRC) and metastasis-free survival (MFS) of the patients were analyzed in relation to αB-crystallin expression. Additionally, the effects of αB-crystallin knockdown on VEGF secretion and cell motility were studied in vitro.

**Results:**

Patients with higher staining fractions of αB-crystallin exhibited a significantly shorter MFS (Log-Rank test, p < 0.005). Under normoxic conditions αB-crystallin knockdown with two different siRNAs in a HNSCC cell line reduced VEGF secretion 1.9-fold and 2.1-fold, respectively. Under hypoxic conditions, a similar reduction of VEGF secretion was observed, 1.9-fold and 2.2-fold, respectively. The effect on cell motility was assessed by a gap closure assay, which showed that αB-crystallin knockdown decreased the rate by which HNSCC cells were able to close a gap by 1.5- to 2.0-fold.

**Conclusions:**

Our data suggest that αB-crystallin expression is associated with distant metastases formation in HNSCC patients. This association might relate to the chaperone function of αB-crystallin in mediating folding and secretion of VEGF and stimulating cell migration.

## Background

The small heat shock protein αB-crystallin (HspB5) is expressed in several types of cancer, including head and neck squamous cell carcinomas (HNSCC) [1,2] and breast carcinomas [3-5]. The expression is often correlated with a poor prognosis, but the reason for this is not fully understood [6,7]. αB-crystallin plays a role in many different cellular processes such as proliferation, cell migration and apoptosis [3,6,8]. The expression of this protein is increased during various stresses, like heat shock and oxidative stress [9]. A well-known function of αB-crystallin is molecular chaperoning, allowing the prevention of aggregation of proteins [9]. Recently, it has been shown that αB-crystallin chaperones the hypoxia-induced VEGF protein to the endoplasmic reticulum, leading to more properly folded and thus secreted VEGF [10,11]. VEGF is a major player involved in tumor angiogenesis [12] and increased VEGF secretion is often correlated with metastasis formation [13] and worse outcome for the patient [14].

HNSCC is the sixth most common cancer worldwide and accounts for 6% of all cancers [15]. The majority of these patients are treated with radiotherapy, alone or in combination with surgery or chemotherapy [16]. Based on the improved understanding of the molecular pathways underlying HNSCC, targeted drugs (e.g. EGFR-specific antibodies) and other modifications have been implemented in treatment protocols [16]. However, only a subset of patients profit from these combined modality strategies. Therefore, there is a great demand for biomarkers to customize treatment.

In the present study the value of αB-crystallin as a biomarker in HNSCC was investigated. αB-crystallin expression levels were immunohistochemically determined in HNSCC biopsies and correlated with clinicopathological characteristics and outcome. Moreover, the effect of knockdown of αB-crystallin on VEGF secretion and cell migration was studied.

## Methods

### Patients

Biopsy material from a cohort of HNSCC patients with stage II to IV primary squamous cell carcinoma of the oral cavity, oropharynx, hypopharynx or larynx was used. The inclusion criteria of patients with HNSCC have been described before [17]. Approval from the ethics committee of Radboud University Nijmegen Medical Centre was obtained and all patients provided written informed consent. Of 13 patients no biopsy materials were left and were excluded from this study. The median duration of follow-up for all patients was 29 months and for surviving patients 85 months. During follow-up, LRC and MFS were registered.

### Immunohistochemical staining of αB-crystallin of HNSCC biopsies

Oral cavity tumor sections [18] were incubated with 100-fold diluted polyclonal rabbit αB-crystallin antiserum [19] and subsequently stained with diaminobenzidine (DAB) according to a standard protocol. Sections of the 38 available biopsies (5 μm) were mounted on poly-L-lysine coated slides, fixed for 10 minutes in acetone at 4°C and rehydrated in PBS. The sections were incubated overnight at 4°C with 100-fold diluted αB-crystallin antiserum [19] and subsequently incubated for 30 minutes at 37°C with 600-fold diluted goat-α-rabbit-FabCy3 (Jackson Immuno Research Laboratories Inc) in PBS, for 45 minutes at 37°C with 10-fold diluted endothelium antibody PAL-E (Euro Diagnostica BV) in PAD, for 60 minutes at 37°C with 100-fold diluted chicken-α-mouse Alexa647 (Molecular probes) in PBS and finally for 5 minutes at room temperature with 0.5 ng/ml Hoechst (Sigma) in PBS. Between the incubation steps, 3 times 2 minutes washing steps in PBS were performed. The sections were mounted using fluorostab (ProGen Biotechnik GmbH).

### Image acquisition

Scanning of the tumor sections was performed with a fluorescence microscope (Axioskop, Zeiss) and a computer-controlled motorized stepping stage, using IP-lab software (Scanalytics)[20]. Each section was completely scanned for αB-crystallin staining. The resulting grey scale images were subsequently binarized. Thresholds were set just above the background staining for each staining. Manually, the total tumor area was contoured, excluding surrounding tissue, large necrotic areas and artefacts. The percentage of αB-crystallin was determined as the tumor area positive for αB-crystallin relative to the total tumor area.

### Cell culture, siRNA treatment, hypoxia exposure and VEGF secretion measurement

The HNSCC cell line, UT-SCC-5 (described in [21]), was maintained in DMEM + Glutamax™ (Invitrogen) supplemented with 10% fetal calf serum (Gibco-BRL) in a standard humidified 37°C incubator_._ At 40% confluency, cells were transfected using Lipofectamine™ 2000 Reagent according to the manufacturers’ protocol (Invitrogen). The siRNAs used were si-Luciferase (siRNA LUC) as negative control, sequence: CGUACGCGGAAUACUUCGAdTdT, si-αB-crystallin1 (siRNA αB1) sequence: GCACCCAGCUGGUUUGACAdTdT and si-αB-crystallin2 (siRNA αB2) sequence: CCCUGAGUCCCUUCUACCUdTdT. After 5 hours, cells were reseeded (9.0x10^3^ cells in 0.3 cm^2^ wells in 12-fold, for VEGF secretion measurements, and 2.5x10^5^ cells in 10 cm^2^ wells in 4-fold, for RNA expression measurements (see below)). Hypoxia treatment was performed 24 hours after siRNA transfection cells in a humidified 37°C H35 Hypoxystation (Don Whitley Scientific) with 0.1% O_2_. Cells cultured under normoxic (6-fold) and hypoxic (6-fold) conditions were maintained for 48 hours. The culture media of the samples were collected and VEGF levels were determined using a quantitative enzyme-linked immunosorbent assay (ELISA). The details of this assay have been described previously by Span and coworkers [22]. The assay is based on the combination of four polyclonal antibodies raised in four different animal species, duck, chicken, rabbit and goat, and are employed in a sandwich assay format. The assay measures VEGF_165_ and VEGF_121_, the main isoforms of VEGF. There is no cross-reactivity with VEGF B, VEGF C and VEGF D [22,23].

### Gap closing assay

The HNSCC cell line UT-SCC-15 [21] was transfected with siRNA as described above. Twenty-four hours after transfection, cells were seeded at 1.0x10^5^ cells per side of the Culture-Inserts (Ibidi, N = 6 per condition). For quantitative RT-PCR, 0.5x10^6^ cells were seeded in parallel in 10 cm^2^ wells (N = 5). 24 Hours later cells were washed with PBS, fresh medium was added and the insert was removed. Time lapse imaging was performed for 24 hours in a microscope stage incubator (Oko-Lab) on a Nikon DiaPhot microscope equipped with a Hamamatsu C8484-05G digital camera. Images were taken every 10 min using TimeLapse Software (Oko-Lab), version 2.7, with a 10x objective. Analysis of gap closing was performed using TScratch [24].

### RNA analysis by quantitative PCR

Total RNA from UT-SCC-5 and UT-SCC-15 cell lysates was extracted using standard Trizol isolation. After DNAse I treatment (Amplification grade, Invitrogen) mRNAs were reverse transcribed using oligo(dT) primers and the Reverse Transcription System (Promega) according to manufacturer’s protocol starting with 1 μg of RNA in a total volume of 20 μl. Subsequently quantitative PCR reactions were performed with 10 μl Power SYBR Green (Applied BioSystems), 5 μM of primers and 2 μl cDNA in a total volume of 20 μl. The sequence of the used αB-crystallin primers is: 5^′^-ATCTTCTTTTGCGTCGCCAG-3^′^ and 5^′^-TTCCCCATGGTGTCTGAGC-3^′^, and of the GAPDH primers: 5^′^-GATTGAGGTGCATGGAAAAC-3^′^ and 5^′^-AGGACCCCATCAGATGACAG-3^′^. The fluorescent signal intensities were recorded with the ABI Prism 7000 system (Applied Biosystems). Samples were kept for 10 minutes at 95°C, followed by 40 cycles of 15 seconds at 95°C and 1 minute at 60°C. Data analysis was performed with 7000 System SDS software (Applied BioSystems).

### Statistics

Statistical analyses were performed using Graphpad Prism 5.00 software. To test for differences in αB-crystallin expression using binary patient data, the unpaired *t*-test was used. For survival analyses, receiver operating characteristic (ROC) curves were made to determine the cut-off value with the highest sensitivity and specificity for discriminating between patients with or without locoregional recurrence (LRR) or distant metastasis with at least 24 months of follow-up or an LRR or distant metastatic event before that. Survival rates were calculated starting at the date of diagnosis. The Kaplan-Meier method and the log-rank test were used to test for differences in LRC and MFS rates in all patients. P-values below 0.05 were considered a priori to indicate a significant difference. Differences in VEGF secretion and gap closure speed were tested using One-way ANOVA and Tukey’s Multiple Comparison Test.

## Results

**Figure 1:**
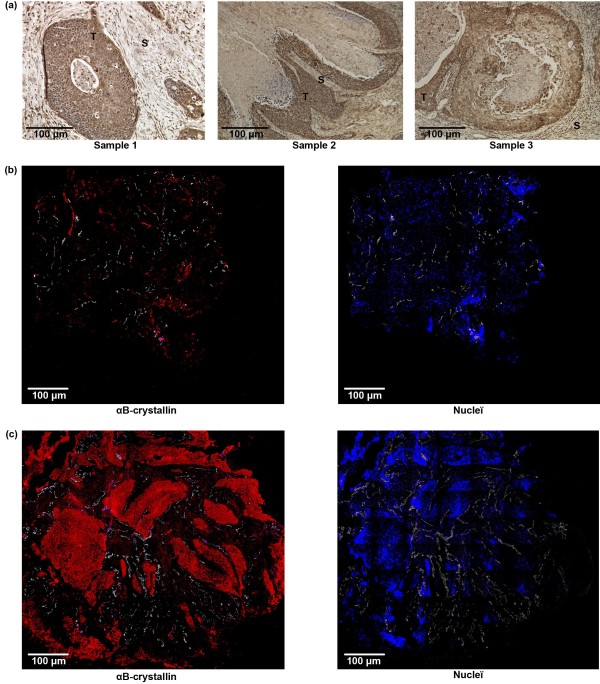
**Immunohistochemical staining of tumor tissues with polyclonal anti-αB-crystallin antibody.** DAB staining of three oral cavity tumor sections. Tumor cells are indicated with T, surrounding stromal cells with S. (**a**). Immunofluorescent staining of biopsies with low αB-crystallin expression (red, **b** left panel) and high αB-crystallin expression (red, **c** left panel) and the corresponding Hoechst stainings (blue, **b** and **c** right panels). Blood vessels are indicated in white (**b** and **c**).

The presence of αB-crystallin in the tumors was analyzed by immunohistological staining. In most tumors cells αB-crystallin could be detected. In stromal cells, no or only very low levels of nuclear staining were found (Figure 1a). The αB-crystallin expression was determined in 38 primary HNSCC biopsies, which were histologically confirmed to contain tumor tissue [17]. The characteristics of the tumors at the time point of biopsy collection are listed in Table 1. To quantify the fraction of the tumor section expressing αB-crystallin, fluorescently labeled secondary antibodies were used and the images were analyzed with a digital image analysis system. Only, the tumor area was used for the analysis by excluding the surrounding stromal tissue, large necrotic areas and artifacts. The results showed that the αB-crystallin expressing tumor areas varied from 0 to 69%. Examples of biopsies having marginal and extensive αB-crystallin expression are shown in Figure 1b and c.

**Table 1:** Characteristics of 38 head and neck squamous cell carcinomas

**Characteristics**	**Number of biopsies**
*Site of tumor*	
Oral cavity	1
Hypopharynx	12
Larynx	15
Oropharynx	10
*T classification*	
1	1
2	13
3	16
4	8
*N classification*	
0	10
1	10
2	18
*M classification*	
0	38
*Differentiation grade*	
1	2
2	21
3	14
Unknown	1

### αB-crystallin expression in HNSCC biopsies is associated with distant metastasis

**Figure 2:**
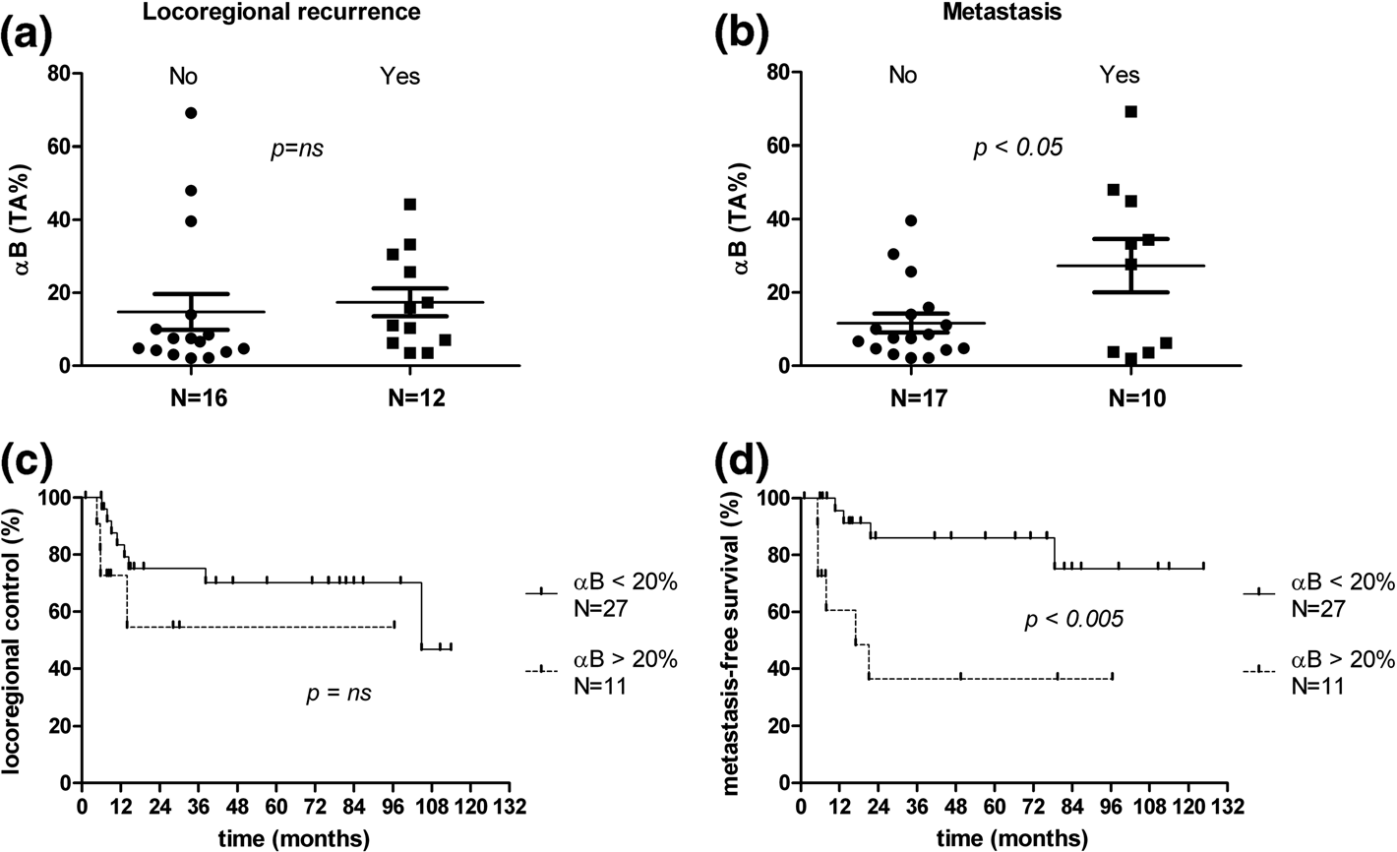
**Relation between αB-crystallin expression and locoregional recurrence and metastasis.** αB-crystallin expression per biopsy and the mean αB-crystallin expression of each group with standard error of the mean (SEM) in biopsies from patients without or with locoregional recurrence (**a**) or metastasis (**b**) and Kaplan-Meier analysis of locoregional control (**c**) and metastasis-free survival (**d**) for patients with high (>20% of tumor area) and low (<20% of tumor area) αB-crystallin expression. TA: Tumor area, ns: not significant.

Patients with at least 24 months of event-free follow up or with a locoregional recurrence (LRR) or distant metastatic event before 24 months were dichotomized in groups with and without LRR (N = 16 and N = 12, respectively), or with and without metastasis (N = 17 and N = 10, respectively). Figure 2 shows that the mean percentage of the tumor area expressing αB-crystallin was not significantly different between biopsies from patients with or without LRR (Figure 2a). However, the mean percentage of the tumor area expressing αB-crystallin was significantly higher in biopsies from patients who developed distant metastasis during follow up as compared to those without (two-tailed unpaired *t*-test, p < 0.05, Figure 2b). The highest sensitivity and specificity for discriminating between patients with or without metastasis was determined by receiver operating characteristic (ROC) curve to be at a cut-off value of 20%. At this cut-off value the Kaplan-Meier estimates for LRC did not show any significant differences (Figure 2c), and the same result was obtained with other cut-off values (results not shown). For MFS a significant difference was observed (Log-Rank, p < 0.005, Figure 2d), which was especially clear after 22 months; at that time point MFS was 86% for the patients with relatively low fractions of tumor area expressing αB-crystallin, whereas this was only 36% for the patients with extensive αB-crystallin expression in the tumors. These results suggest that the levels of αB-crystallin expression inversely correlate with MFS.

### αB-crystallin expression enhances VEGF secretion

**Figure 3:**
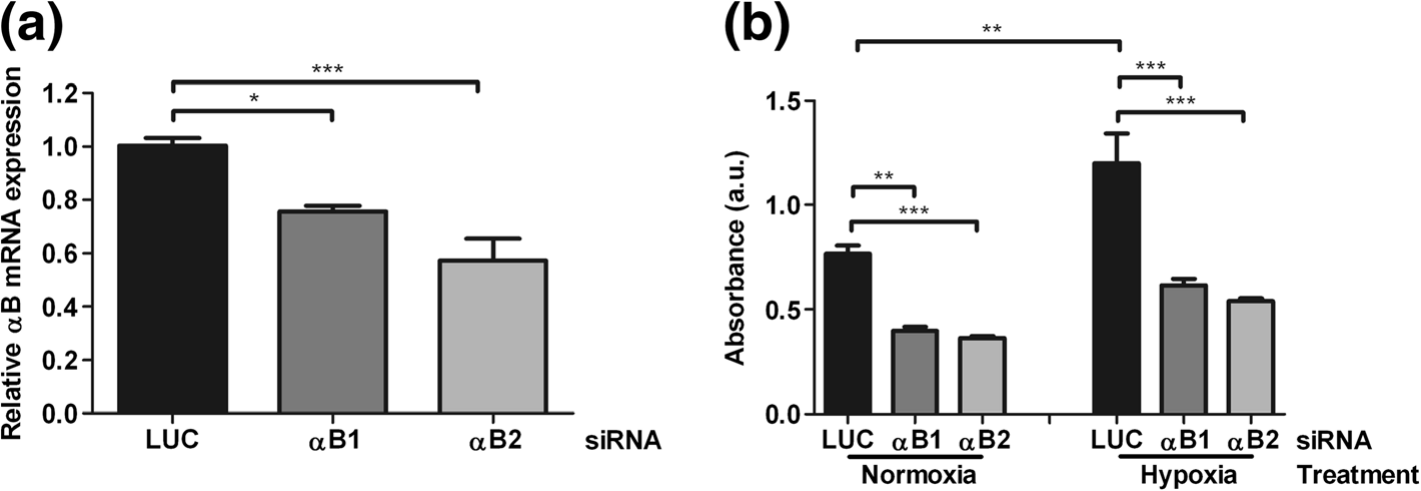
**Influence of αB-crystallin knock-down on VEGF secretion.** Knockdown of αB-crystallin mRNA expression in UT-SCC-5 cells by two different siRNAs, αB1 and αB2, compared to negative control siRNA LUC (Luciferase) as measured by quantitative PCR (**a**). VEGF secretion levels in UT-SCC-5 cells transfected with the αB1, αB2 or LUC siRNA under normoxic and hypoxic conditions determined by ELISA (**b**). Statistical analysis was performed using One-way ANOVA and Tukey’s Multiple Comparison Test. *** P < 0.001, ** 0.001 < P < 0.01, * 0.01 < P < 0.05; a.u: absorbance units.

The recently reported involvement of αB-crystallin in VEGF production prompted us to investigate the effects of αB-crystallin expression on VEGF secretion by HNSCC cells. For these experiments the HNSCC cell line UT-SCC-5 was used and the levels of αB-crystallin were reduced by siRNA-mediated knock-down. Two distinct αB-crystallin siRNAs, αB1 and αB2, resulted in a 1.3-fold and 1.8-fold reduction of αB-crystallin mRNA levels, respectively (Figure 3a). Downregulation of αB-crystallin significantly decreased VEGF secretion (1.9-fold and 2.1-fold reduction, respectively). Since hypoxia leads to elevated VEGF production, we next assessed the effects of αB-crystallin knock-down under hypoxic conditions. Indeed, VEGF secretion appeared to be increased in UT-SCC-5 cells by hypoxia (Figure 3b, 1.6-fold) and also under these conditions αB-crystallin depletion resulted in decreased VEGF expression (1.9-fold and 2.2-fold reduction, respectively). These results show that αB-crystallin expression can affect VEGF secretion both at normoxia and hypoxia.

### Effect of αB-crystallin expression on cell migration

**Figure 4:**
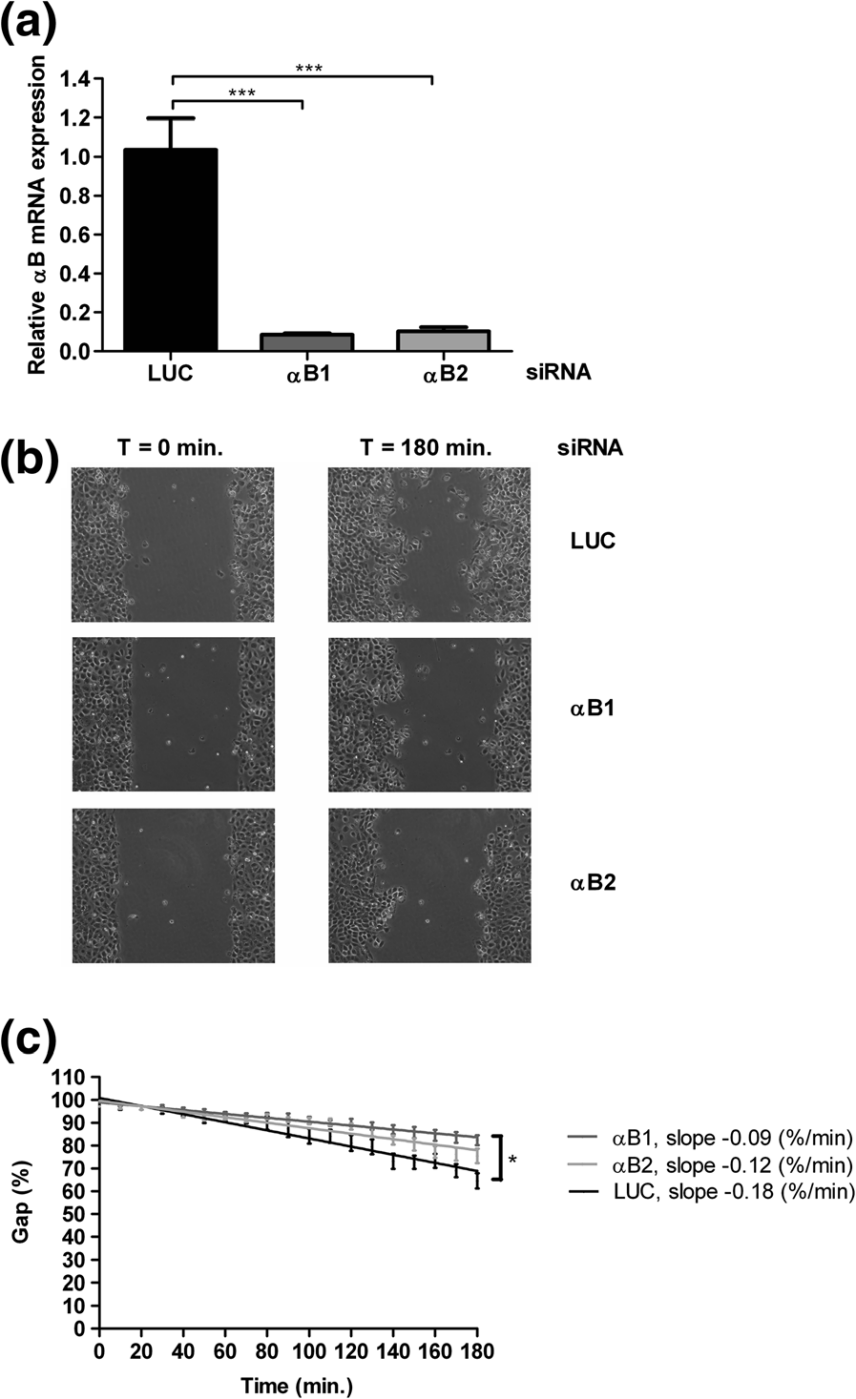
**Influence of αB-crystallin knock-down on cell migration determined with gap closure assay.** Knockdown of αB-crystallin mRNA expression in UT-SCC-15 cells by two different siRNAs, αB1 and αB2, compared to negative control siRNA LUC (Luciferase) as measured by quantitative PCR (**a**). Representative micrographs of the gap closure by UT-SCC-15 cells after 0 and 180 minutes at 10x magnification. (**b**). Relative gap size between 0 and 180 minutes of UT-SCC-15 cells transfected with the αB1, αB2 or LUC siRNA (**c**). Statistical analysis was performed using One-way ANOVA and Tukey’s Multiple Comparison Test. *** P < 0.001, * 0.01 < P < 0.05.

The association of αB-crystallin expression with metastasis may also be due to effects on cell motility [3]. To investigate whether motility of HNSCC cells is affected by reduced αB-crystallin a gap closure assay was applied. Since the UT-SCC-5 cells were easily damaged during gap preparation, disturbing migration, the related cell line UT-SCC-15 was used. Also in this HNSCC cell line αB-crystallin mRNA levels could be diminished by siRNA-mediated knock-down, although the efficiency appeared to be much higher than that in the UT-SCC-5 cells (Figure 4a). Depletion of αB-crystallin resulted in decreased gap closure rates compared to mock treated cells (Figure 4b). The cell migration rate was reduced 2.0-fold from and 1.5-fold after treatment with the siRNA αB1 and αB2, respectively (Figure 4c).

## Discussion

Here, we show that αB-crystallin expression in HNSCC tumors correlates with MFS, but not with LRC. Clues for the mechanism by which αB-crystallin might affect distant metastasis formation in patients were obtained by the depletion of αB-crystallin in HNSCC cell lines, which showed that both VEGF secretion and cell motility were decreased when αB-crystallin expression was reduced.

Previously, two studies have addressed the prognostic value of αB-crystallin in HNSCC. Chin and coworkers have shown that αB-crystallin is a marker for poor prognosis [1], which is substantiated by our data. However, they noted that none of the patients lacking αB-crystallin had LRR, while 37% of the patients with tumors stained positive for αB-crystallin had LRR. In the current study, the difference in prognosis was mainly caused by a higher rate of distant metastasis in the high αB-crystallin group and not by a difference in LRR. Boslooper and colleagues did not find αB-crystallin to be a prognostic marker for HNSCC [2]. These researchers observed the highest concentrations of αB-crystallin in the centre of tumor cell nests and not the more diffuse localization observed in this study and the study of Chin and coworkers [1]. This difference in αB-crystallin localization may have been caused by a difference in staining procedure and could be the reason why this study led to a different conclusion.

αB-crystallin was also found to be associated with a poor prognosis in several other types of cancers, such as breast cancer [3-5,25,26] and was downregulated in a breast cancer cell line by breast cancer metastasis suppressor 1, which specifically suppresses metastasis [27]. αB-crystallin is a molecular chaperone able to prevent protein aggregation and can confer protection to cells under stress conditions. αB-crystallin inhibits apoptosis in response to different anti-cancer agents, such as DNA-damaging drugs, TNFα and Fas ligand [6] and has been shown to be a predictor of resistance to chemotherapy [28]. However, since our data do not support an association of αB-crystallin expression with local recurrence after treatment of the patients, the cytoprotective activity is likely not the sole reason why αB-crystallin is associated with poor prognosis.

Metastasis formation occurs via a series of steps, also known as the “metastatic cascade” [29]. One of the factors associated with metastatic spread is VEGF [30,31]. Several studies have demonstrated roles for αB-crystallin in VEGF-dependent angiogenesis [32]. Tumor vasculature in αB-crystallin-deficient mice displays high levels of endothelial apoptosis and decreased vessel formation. αB-crystallin has been shown to associate with VEGF-A (the most well-known member of the VEGF protein family) [8,33] and colocalizes with VEGF at the endoplasmic reticulum [11]. Furthermore, VEGF-A expression remained low in αB-crystallin-deficient mice during retinal revascularization after artificially-induced retinopathy [10]. Here we have shown that in a HNSCC cell line αB-crystallin is also involved in secretion of VEGF and in this way may influence tumorigenic blood vessel formation and metastasis formation in HNSCC. For the growth of metastatic tumors in lymph nodes neoangiogenesis is not absolutely needed [34,35], which might explain why no correlation was found between αB-crystallin expression and LRC.

Migration-associated proteins may also correlate with worse outcome, as has been shown for patients with squamous cell carcinoma of the tongue [36]. αB-crystallin can affect cell migration as well. Overexpression of αB-crystallin led to higher cell motility in several studies [3,25,37]. Here we have shown that depletion of αB-crystallin decreased the motility of UT-SCC cells, indicating also in these types of cells αB-crystallin may affect cell migration. VEGF also plays a role in cell migration [38], potentially by enhancing invadopodia formation [39]. The effect of αB-crystallin on cell migration may thus be mediated by VEGF, although other mechanisms are also possible, as for example by the influence of αB-crystallin on actin filaments dynamics [39,40]. Further research is needed to reveal the molecular mechanisms by which αB-crystallin affects cell migration.

## Conclusions

High αB-crystallin expression is associated with metastasis formation in HNSCC but not with locoregional recurrence. αB-crystallin could be a useful biomarker to help fine-tune treatment, possibly by targeting αB-crystallin-induced VEGF secretion or cell motility. Validation in a larger HNSCC cohort is required to confirm the significance of this finding.

## Abbreviations

HNSCC: Head and neck squamous cell carcinoma; LRC: Locoregional control; LRR: Locoregional recurrence; MFS: Metastasis-free survival; ROC: Receiver operating characteristic; VEGF: Vascular endothelial growth factor

## Competing interests

The authors have no competing interest to declare.

## Authors’ contributions

CS participated in the study concept and design, data acquisition of all figures, data analysis and interpretation, statistical analysis, manuscript preparation and editing. JB participated in the study concept and design and manuscript editing. PS participated in study design, data analysis and interpretation, statistical analysis and manuscript editing. FS participated in data acquisition of the VEGF figure and manuscript reviewing. RG participated in data acquisition of the VEGF and cell motility figure and in manuscript reviewing. HS participated in data acquisition of the VEGF and cell motility figure. GP participated in study concept and manuscript reviewing. JK participated in study concept and design and manuscript editing. WB participated in study concept and design, data analysis and interpretation and manuscript editing. All authors read and approved the final manuscript.

## Pre-publication history

The pre-publication history for this paper can be accessed here:

http://www.biomedcentral.com/1471-2407/13/128/prepub
